# Reversible tuning of membrane sterol levels by cyclodextrin in a dialysis setting

**DOI:** 10.1016/j.bpj.2025.03.020

**Published:** 2025-03-25

**Authors:** Cynthia Alsayyah, Emmanuel Rodrigues, Julia Hach, Mike F. Renne, Robert Ernst

**Affiliations:** 1 Medical Biochemistry and Molecular Biology, Medical Faculty, Saarland University, Homburg, Saar, Germany; 2 Preclinical Center for Molecular Signaling (PZMS), Medical Faculty, Saarland University, Homburg, Saar, Germany; 3 Center for Biophysics (ZBP), Saarland University, Saarland, Germany

## Abstract

Large unilamellar vesicles are popular membrane models for studying the impact of lipids and bilayer properties on the structure and function of transmembrane proteins. However, the functional reconstitution of transmembrane proteins in liposomes can be challenging, especially if the hydrophobic thickness of the protein does not match the thickness of the lipid bilayer. Such hydrophobic mismatch causes protein aggregation and low yields during the reconstitution procedure, which are exacerbated in sterol-rich membranes featuring low membrane compressibility. Here, we explore new approaches to reversibly tune the sterol content of (proteo)liposomes with methyl-*β*-cyclodextrin (m*β*CD) in a dialysis setting. Maintaining (proteo)liposomes in a confined compartment minimizes loss of material during cholesterol transfer and facilitates efficient removal of m*β*CD. We monitor the sterol concentration in the membrane with help of the solvatochromic probe C-Laurdan, which reports on lipid packing. Using Förster resonance energy transfer, we show that cholesterol delivery to proteoliposomes induces the oligomerization of a membrane property sensor, whereas a subsequent removal of cholesterol demonstrates full reversibility. We propose that tuning membrane compressibility by m*β*CD-meditated cholesterol delivery and removal in a dialysis setup provides a new handle to study the impact of sterols and membrane compressibility on membrane protein structure, function, and dynamics.

## Significance

Generating complex, sterol-rich, biomimetic membranes for studying the structure and function of reconstituted membrane proteins is challenging. As an important step toward asymmetric, sterol-rich, complex model membrane systems, we have established a procedure to control the membrane sterol levels of liposomes and proteoliposomes using methyl-*β*-cyclodextrin in a dialysis setup. We gain control over the membrane sterol content and follow sterol transfer by C-Laurdan and dehydroergosterol fluorescence spectroscopy. We explore several parameters that affect the rate of cholesterol transfer, demonstrate applicability for biomembranes, and show that the oligomerization of the membrane property sensor Ire1, which regulates the unfolded protein response in yeast, is controlled by the cholesterol content of the surrounding lipid bilayer.

## Introduction

Biological membranes possess collective biophysical properties such as fluidity, thickness, and compressibility, which influence the structure, oligomeric state, and function of membrane proteins (1,2,3,4). Cholesterol is one of the most abundant lipids in mammalian cells and crucially important for modulating bilayer properties. It is asymmetrically distributed across the plasma membrane and regulates membrane permeability, membrane stiffness, and phase behavior (5,6,7,8,9,10). By increasing lipid packing, it also increases the hydrophobic thickness of the lipid bilayer and reduces membrane compressibility (11).

When the transmembrane domain of a protein does not match the hydrophobic thickness of the surrounding bilayer, it can be driven into oligomers to minimize the energetic strain from lipid distortion (1,12,13,14). Lipid scramblases and membrane protein insertases, on the other hand, induce membrane thinning to facilitate lipid exchange between the two leaflets and/or to move hydrophilic sections of transmembrane client proteins through the hydrophobic membrane core (4,15,16). These examples provide evidence for an important role of membrane compressibility in regulating membrane protein function (4). However, it remains challenging to quantify the contribution of individual membrane properties to a specific transmembrane protein function for at least three reasons. Firstly, the collective bilayer properties are physically connected and interdependent. This makes it challenging to modulate one property without perturbing others (17). Secondly, most biophysical properties of biological membranes can neither be measured directly nor deduced from their (often unknown) composition (18). Thirdly, it remains challenging to reconstitute membrane proteins in complex, asymmetric, and sterol-rich membranes for a functional characterization under defined conditions. This is particularly true for proteins such as lipid scramblases or membrane property sensors that rely on membrane distortions and hydrophobic mismatch for their functions. Reconstituting proteins with a substantial hydrophobic mismatch in cholesterol-rich membranes causes protein aggregation, low yields, and a heterogeneous distribution of proteins and lipids in the preparation (19,20,21,22). This challenge is further complicated by phase-separation phenomena, in which cholesterol contributes to lateral inhomogeneities in the membrane. Hence, there is an urgent need for new experimental paradigms that facilitate the characterization of isolated transmembrane proteins in biomimetic, sterol-rich, defined membrane environments.

Here, we make a first step in this direction. We use methyl-*β*-cyclodextrin (m*β*CD) in a dialysis setup to modulate the cholesterol content of preformed (proteo)liposomes (23,24,25,26). M*β*CD is a ring-shaped, hydrophilic oligosaccharide with a hydrophobic cavity (6–6.5 Å) that is sufficiently large to accommodate cholesterol, thereby making it a perfect, water-soluble shuttle that can either deliver or remove sterols (27,28). Using cholesterol-loaded m*β*CD in a dialysis setup maintains the (proteo)liposomes in a confined compartment while allowing facile sterol transfer. Furthermore, the setup provides a means for a straightforward buffer exchange and quantitative removal of m*β*CD after tuning the sterol concentration. We follow cholesterol insertion into liposomes quantitatively using C-Laurdan spectroscopy (29), determine the rate of delivery, and use m*β*CD to manipulate the sterol levels of microsomes isolated from cells. We demonstrate the cholesterol-dependent, membrane-based dimerization of the membrane property sensor Ire1, which monitors membrane compressibility by a hydrophobic mismatch-based mechanism (30,31,32). Hence, we expand the applications of m*β*CD in membrane research by the implementation of a dialysis setup for the directed and controlled manipulation of (proteo)liposomes and biomembranes.

## Materials and methods

### Production of multilamellar vesicles

Liposomes of defined compositions were generated by mixing 1,2-dioleoyl-*sn*-glycero-3-phosphocholine (DOPC),1-palmitoyl-2-oleoyl-*sn*-glycero-3-phosphocholine (POPC), and cholesterol (25 mg/mL stock) or dehydroergosterol (DHE) (1 mg/mL stock) in chloroform. Those were 1) 100% POPC; 2) 50 mol % DOPC, 50 mol % POPC; 3) X mol % cholesterol (100-X) mol % POPC; 4) X mol % cholesterol (50-X/2) mol % POPC (50-X/2) mol % DOPC; and 5) X mol % DHE (100-X) mol % POPC. Typically, we prepared multilamellar vesicles in batches containing 100 *μ*mol of lipids in 1 mL. The solvent was evaporated under a stream of nitrogen in a heating block at 60°C. For a complete removal of the solvent, the lipid film was subjected to high vacuum in a desiccator for at least 1 h at 23°C ± 1°C. The lipid cake was then rehydrated with pre-warmed liposome buffer (20 mM HEPES pH 7.4, 150 mM NaCl, 5% (w/v) glycerol) to reach the desired lipid concentration and for forming multilamellar vesicles. The liposome suspensions were agitated in a thermal mixer (60°C; 1200 rpm; 30 min) and then sonicated in a water bath for 20 min at 60°C and at power setting 9 (VWR ultrasonic cleaner). The suspension of multilamellar vesicles was used for protein reconstitution experiments at 23°C ± 1°C or snap-frozen with liquid nitrogen and stored at −80°C.

### Preparation of extruded liposomes

The multilamellar vesicles were subjected to seven cycles of freeze-thawing using liquid nitrogen for freezing and a water bath at 40°C for thawing. Next, the suspension was extruded 31 times using a polycarbonate membrane with 200-nm pore size. Due to the absence of anionic lipids in our formulations, it is likely that the resulting liposomes are multilamellar to some extent (33).

### Liposome leakiness assay

5(6)-Carboxyfluorescein (CF)-loaded liposomes were prepared by rehydrating a lipid film (100 mol % POPC) in inside buffer (20 mM HEPES, 75 mM CF, pH 7.4) followed by seven cycles of freeze-thawing and extrusion though a 200-nm membrane. The suspension or CF-loaded liposomes was loaded onto a PD-10 column to remove CF outside the liposomes. For measuring the fluorescence of CF-loaded liposomes, a 20-*μ*L suspension (4 nmol of lipid) of CF-loaded liposomes (200 *μ*M) was diluted 10-fold in 200 *μ*L of liposome buffer in a 96-well plate, and the fluorescence was measured using a TECAN SPARK 20M plate reader (Excitation (Ex.), 492 ± 5 nm; Emission (Em.), 517 ± 5 nm) over time at 30°C. After the treatment of the liposomes with 0.88 *μ*L of either a cholesterol-loaded m*β*CD solution (50 mM in PBS) or an empty m*β*CD solution (100 mM in PBS) yielding a final concentration of 220 and 440 *μ*M respectively, the CF fluorescence was observed over time. As control, liposome buffer (20 mM HEPES pH 7.4, 150 mM NaCl, 5% (w/v) glycerol) was added to a separate well with a liposome suspension. The addition of Triton X-100 to a final concentration of 0.1% (w/v) releases all CF by solubilizing the liposomes.

### Preparation of cholesterol-loaded m*β*CD

Cholesterol-loaded m*β*CD was prepared as described previously (34). Briefly, 132 mg of m*β*CD (100 *μ*mol) and 11.9 mg of cholesterol (30.8 *μ*mol) were dissolved in 600 *μ*L methanol by rigorous mixing at 23°C ± 1°C. The solvent was evaporated under a stream of nitrogen. For a full removal of the solvent, the cholesterol-loaded m*β*CD was transferred in a desiccator and subjected to vacuum for 1 h. The resulting material was resuspended in 2 mL of PBS at a final m*β*CD concentration of 50 mM and subjected to sonification in a water bath (37°C, full power, 3 min) and then incubated in a shaker at 37°C overnight.

### Cholesterol delivery and removal setup

1 mL of 50 mM cholesterol-loaded m*β*CD is diluted 21-fold in liposome buffer (20 mM HEPES pH 7.4, 150 mM NaCl, 5% (w/v) glycerol) to yield the final m*β*CD of ≈2.4 mM in 21 mL. For cholesterol removal, 1 mL of empty m*β*CD (100 mM) is used equivalently to yield 21 mL of m*β*CD at a concentration of ≈4.8 mM. Before use, Spectra-Por Float-A-Lyzer G2 cassettes (100 kDa) are hydrated in liposome buffer. They are then placed in the outer bath (V = 21 mL) containing liposome buffer with either cholesterol-loaded or empty m*β*CD. 1 mL of acceptor (proteo)liposomes are pipetted in the dialysis cassette (for liposomes 200 *μ*mol lipids; for proteoliposomes 600 *μ*mol lipids assuming 100% recovery during membrane protein reconstitution). The outer bath is stirred with a magnetic stirrer at 270 rpm (VELP Scientifica-F203A0178). The dialysis setup was protected from light whenever fluorescent molecules were used.

### C-Laurdan spectroscopy

To measure lipid packing, we used the solvatochromic dye 6-dodecanoyl-2-[N-methyl-N-(carboxymethyl)amino]naphthalene (C-Laurdan) (29) (Bio-Techne, 7273). A working solution of C-Laurdan (20 *μ*M) was prepared from a 2 mM stock solution in DMSO by 100-fold dilution in liposome buffer (20 mM HEPES pH 7.4, 150 mM NaCl, 5% (w/v) glycerol) and typically used up within 1 h. 60 *μ*L of a liposome suspension (12 nmol lipid) was mixed with 188.8 *μ*L of liposome buffer in a 96-well plate. A scattering control was recorded under identical conditions as for C-Laurdan fluorescence spectroscopy (Ex., 375 ± 5 nm; Em., 400–530 nm; Em. slit width, 5 nm) in a TECAN SPARK 20M plate reader at 30°C. After this, 1.2 *μ*L of the 20 *μ*M C-Laurdan working solution was added to the liposome-containing sample to yield a final C-Laurdan concentration of 96 nM and a final lipid concentration of 48 *μ*M in a total volume of 250 *μ*L (C-Laurdan:glycerophospholipid ratio of 1:500). Hence, the concentration of DMSO in the final sample was 0.005% (v/v). After 5 min of incubation at 30°C, the C-Laurdan fluorescence emission spectrum was recorded and corrected by subtracting the scattering control. The generalized polarization (GP) was calculated from the corrected spectrum by integrating the intensities between 400 and 460 nm (*I*_Ch1_), and 470 and 530 nm (*I*_Ch2_) and using the following equation:

(1) $$\text{GP}=\frac{\left(I\text{Ch}1-I\text{Ch}2\right)}{\left(I\text{Ch}1+I\text{Ch}2\right)}$$

### Dynamic light scattering

100 *μ*L of liposome suspensions (2 nmol of lipids) other samples in liposome buffer were placed in quartz microcuvettes (3 × 3 mm; 8.5 mm; 105.251-QS, Hellma Analytics, Germany) and analyzed using a Zetasizer Nano S (Malvern Panalytical, Worcestershire, UK) for their dynamic light scattering (DLS) after 60 s of equilibration at 25°C with the settings RI = 1.45 and at Abs = 0.001. All samples were measured three times (11 runs of 10 s per measurement), with the attenuator position automatically optimized for each measurement. Data analysis was performed using the Zetasizer software version 7.13.

### DHE exchange experiments

1.5 mL of an empty m*β*CD (stock solution of 100 mM) was diluted 14-fold in liposome buffer (20 mM HEPES pH 7.4, 150 mM NaCl, 5% (w/v) glycerol) to yield a 21-mL outer bath at an initial m*β*CD concentration of 7.14 mM. Before use, Spectra-Por Float-A-Lyzer G2 cassettes (100 kDa) are hydrated in liposome buffer. 1 mL of a suspension POPC-based liposomes containing 2 mol % DHE (200 nmol total lipid) were pipetted into the dialysis cassette with a 100-kDa molecular-weight cutoff. The outer bath contained nonfluorescent, POPC-based acceptor liposomes (400 nmol total lipid) and 7.14 mM m*β*CD. Before starting the lipid exchange, less than 1% of the total sample was retrieved from the dialysis cassette for characterizing the DHE content by fluorescence spectroscopy. After 24 h of lipid exchange at 23°C ± 1°C, less than 1% of the total sample was retrieved from both the inner and outer compartment (5.7 *μ*L from the dialysis cassette and 60 *μ*L from the outer bath). All samples were adjusted to 120 *μ*L using liposome buffer (20 mM HEPES pH 7.4, 150 mM NaCl, 5% (w/v) glycerol) for subsequent measurement. After exchange, the dialysis cassette with donor liposomes was placed in a new dialysis bath and dialyzed against 300 mL of liposome buffer to remove all DHE, which is not associated with liposome membranes. In parallel, 1 mL of the outer bath was retrieved, placed into a fresh dialysis cassette (100-kDa molecular-weight cutoff) dialyzed against 300 mL of liposome buffer. Following dialysis, a 5.7-*μ*L sample was retrieved from the inside of the donor cassette and 60 *μ*L from the acceptor cassette. All samples were adjusted to 120 *μ*L using liposome buffer (20 mM HEPES pH 7.4, 150 mM NaCl, 5% (w/v) glycerol) and subjected to a quartz cuvette (10 × 2 mm; 15 mm; 105.250-QS, Hellma Analytics, Germany). DHE was quantified using its fluorescence emission (Ex., 324 ± 4 nm; Em., 394 ± 4 nm) in a FluoroMax 4 (Horiba, Japan) at 25°C. All fluorescence data were corrected for scattering using control spectra using extruded liposomes with identical lipid composition and concentration, but lacking DHE.

### Isolation of crude microsomes from HEK293T cells

HEK293T cells were cultivated in 10 × 15-cm cell culture dishes to ∼80% confluency before harvesting and washing them with 5 mL of cold PBS per plate. Cells were pelleted by centrifugation (500 × *g*, 5 min, 4°C) before adding 10 cell volumes of hypotonic buffer (20 mM HEPES pH 7.5, 5 mM KCl, 1.5 mM MgCl_2_, 2 mM DTT, 0.03 mg/mL protease inhibitor cocktail). The cells were incubated on ice for 15 min and occasionally agitated. Cells were lysed by 35 strokes in a pre-cooled glass homogenizer with a tightly fitting pestle on ice. The lysate was mixed with 2.5 volumes of membrane buffer 1 (20 mM HEPES pH 7.5, 525 mM mannitol, 175 mM sucrose, 5 mM EDTA, 2 mM DTT, 0.03 mg/mL protease inhibitor cocktail) by gentle inversion. Unbroken cells, nuclei, and debris were removed by two steps of centrifugation (700 × *g*, 10 min, 4°C). The final supernatant fraction was centrifuged twice (10,000 × *g*, 10 min, 4°C) to yield a crude microsome fraction in the pellet. The crude microsome fraction was resuspended in ∼300 *μ*L of membrane buffer 2 (20 mM HEPES pH 7.5, 210 mM mannitol, 70 mM sucrose, 0.5 mM EDTA, 2 mM DTT, 0.03 mg/mL protease inhibitor cocktail), aliquoted into portions of 50 *μ*L, frozen with liquid nitrogen, and stored at −80°C. Before each use, crude microsome aliquots were thawed on ice and sonicated for 10 s (50% volume as 70% pulses) with a tip sonifier (Sonotrode MS72; Bandelin Sonopuls HD 2070).

### Thin-layer chromatography and lipid staining

Lipids were extracted using the two-step Bligh and Dyer extraction (35) with minor changes. Briefly, 100*μ*L of sample was mixed with 100 *μ*L of ammonium bicarbonate solution (150 mM). After the addition of 750 *μ*L of chloroform:MeOH (2:1), the sample was vigorously shaken for 15 min at 23°C ± 1°C. A subsequent addition of 250 *μ*L of chloroform and 250 *μ*L of ammonium bicarbonate (150 mM) induced phase separation. Again, the sample was vigorously agitated for 15 min. The organic phase was collected after centrifugation (2000 × *g*, 2 min). The aqueous phase was subjected to another round of lipid extraction after the addition of 500 *μ*L of chloroform. The organic phases were pooled. The lipids was dried for at least 1 h in a speedvac (Thermo Fisher Scientific, Savant DNA110) and taken up 10 *μ*L of chloroform. Lipid separation on a thin-layer chromatography (TLC) Silica 60 gel layer ADAMANT (Macherey-Nagel) was performed using chloroform:methanol:H_2_O at a 70:25:2 ratio (v/v) as the mobile phase. After migration, the silica plates were dried in a chemical hood for 15 min, and lipids were stained for 10–15 min with iodine. The silica plates were scanned on an EPSON V750 PRO and analyzed using Fiji (Fiji version 2.16.0) (36).

### Bacterial cultivation for protein purification

*Escherichia coli* BL21 pLysS carrying the expression vector (pRE982) encoding for a fusion protein of the maltose-binding protein (MBP) and the transmembrane region of Ire1^P501-K570, C552S^ from *Saccharomyces cerevisiae* with a C-terminal cysteine for labeling via maleimide chemistry were cultivated in 50 mL of lysogeny broth (LB) medium (containing ampicillin and chloramphenicol) at 37°C for 16–18 h under continuous shaking (220 rpm). The overnight culture was used to inoculate 2 L of LB medium supplemented with 0.2% glucose, ampicillin, and chloramphenicol to an optical density 600 of 0.05 at 37°C. The expression was induced with 0.3 mM Isopropyl-β-D-1-thiogalactopyranosid (IPTG) at an optical density 600 of 0.6. After 3 h of induction, the cells were harvested by centrifugation (3000 × *g*, 20 min, 4°C), washed with ice-cold column buffer (50 mM HEPES pH 7.0, 150 mM NaCl, 1 mM EDTA, pH 8.0), and centrifuged again (3000 × *g*, 20 min, 4°C). The resulting cell pellets were stored at −20°C.

### Extraction, purification, and labeling of a membrane property sensor

Typically, a cell pellet from a 2 L bacterial culture was thawed on ice. Cells were resuspended in 45 mL of ice-cold lysis buffer (50 mM HEPES pH 7.0, 150 mM NaCl, 1 mM EDTA pH 8.0, 50 mM 1-O-*n*-octyl-*β*-D-glucopyranoside (OG)) containing 10 mg/mL chymostatin, 10 mg/mL antipain, 10 mg/mL pepstatin, 10 mM TCEP, 25 units/mL Benzonase (Merck). The suspension was sonified using a VS 70T probe and SONOPULS HD 2070 (Bandelin) at 30% power and with 70% duty in six cycles of 30 s of sonication and 30 s of intermission. The resulting lysates were rotated for 30 min at 4°C. Cell debris was removed by ultracentrifugation (100,000 × *g*, 30 min, 4°C) using a Type 70 Ti rotor (Beckmann Coulter). The supernatant was transferred onto pre-equilibrated amylose beads in column buffer (50 mM HEPES pH 7.0, 150 mM NaCl, 1 mM EDTA-NaOH pH 8.0) and incubated under constant agitation for 60 min. The suspension of amylose beads was then distributed to two gravity columns. Each column was washed twice with 20 mL of degassed lysis buffer (50 mM HEPES pH 7.0, 150 mM NaCl, 1 mM EDTA pH 8.0, 50 mM OG). Next, 1.2 mL of labeling solution containing either ATTO514, ATTO594, or NEM at a final concentration of 0.25 mM in lysis buffer (50 mM HEPES pH 7.0, 150 mM NaCl, 1 mM EDTA pH 8.0, 50 mM OG) was added. Cysteine modification took place during an overnight incubation at 4°C under constant agitation. Each column was washed three times with 20 mL of lysis buffer to remove unreacted ATTO dyes or NEM. The labeled protein was eluted in three steps using each 2 mL of elution buffer (50 mM HEPES pH 7.0, 150 mM NaCl, 1 mM EDTA pH 8.0, 50 mM OG, 10 mM maltose, 10% (w/v) glycerol). Each time, the elution buffer was incubated for 5 min on the column. The pooled eluate was concentrated to a final volume of 600 *μ*L using a spin concentrator with a 30-kDa molecular-weight cutoff, snap-frozen in liquid nitrogen, and stored at −80°C for later use. For further purification, the protein was subjected to size exclusion chromatography. A protein aliquot was thawed at 23°C ± 1°C and centrifuged (20,000 × *g*, 10 min, 4°C) to remove potential protein aggregates. 500 *μ*L from the supernatant were loaded at a flow rate of 0.5 mL min^−1^ onto a Superdex Increase 200 column equilibrated with gel filtration buffer (20 mM HEPES pH 7.4, 150 mM NaCl, 50 mM OG). Protein-containing fractions were pooled and adjusted to a final glycerol concentration of 10% (w/v).

### Reconstitution of a membrane property sensor in liposomes

For each reconstitution, 200 *μ*L of multilamellar vesicles (from a 10 mM stock) were mixed with 20 mM HEPES pH 7.4, 150 mM NaCl, and 37.5 mM OG and agitated on a rotor for 10 min for complete lipid solubilization. 3.3 *μ*g of fluorescent (and nonfluorescent) proteins (in 20 mM HEPES pH 7.4, 150 mM NaCl, 50mM OG, 10% (w/v) glycerol) were added yielding an overall protein-to-lipid ratio of 1:16,000. Glycerol and SDS were adjusted to reach a final concentration of 7% (w/v) and 0.3 mM, respectively, in a total volume of 1 mL of reconstitution mix (20 mM HEPES pH 7.4, 150 mM NaCl, and 37.5 mM OG). After 10 min of agitation, the mix was transferred in a dialysis cassette with a 10-kDa molecular-weight cutoff and dialyzed against 1 L of dialysis buffer (20 mM HEPES pH 7.4, 150 mM NaCl, 5% (w/v) glycerol). 400 mg of methanol-activated, washed, and equilibrated SM-2 Bio-Beads were added to the outer bath of the dialysis setup to provide a sink for detergent molecules. After 1 h, the dialysis cassette was placed in a new bath with 1 L of dialysis buffer (without SM-2 Bio-Beads) and dialyzed for 1 h. This step was repeated twice. As the final step, the cassette was dialyzed overnight against dialysis buffer with 800 mg of methanol-activated, washed, and equilibrated SM-2 Bio-Beads to remove the last traces of OG and to yield proteoliposomes with the Ire1-based membrane property sensor.

### Determining the relative Förster resonance energy transfer efficiency

Liposomes containing different combinations of unlabeled (NEM) and labeled (ATTO514 or ATTO594) version of MBP-Ire1^P501-K570, C552S^ with a C-terminal cysteine were used to determine the relative Förster resonance energy transfer (FRET) efficiency in proteoliposomes containing both the fluorescence donor (ATTO514) and acceptor (ATTO594) construct. The protein-to-lipid ratio was 1:16,000 in each case. Fluorescence emission spectra (Ex., 514 ± 3 nm; Em., 512–800 nm; Em. slit width, 3 nm) were recorded in proteoliposomes buffer (20 mM HEPES pH 7.4, 150 mM NaCl, 7% (w/v) glycerol) with an integration time to 0.1 s (FluoroMax 4, Horiba, Japan) from 120-*μ*L samples (0.6 mM lipid) in a 10 × 2-mm quartz cuvette (105.250-QS, Hellma Analytics, Germany) at 30°C. A second spectrum was recorded after solubilizing the proteoliposomes with detergent solution (20 mM HEPES pH 7.4, 150 mM NaCl, 7% (w/v) glycerol, 50 mM OG, 4 mM SDS). As the bleed-through for both the donor and acceptor fluorescence was low, and because only semi-quantitative information was required, we determined a ratiometric FRET (relative FRET: *E*_rel_). The fluorescence spectra were normalized to the highest fluorescence emission (around 535 nm). Normalized spectra from a sample containing only the donor Ire1^ATTO514^ construct were subtracted from normalized spectra containing both Ire1^ATTO514^ and Ire1^ATTO594^ (donor and acceptor).

The relative FRET efficiency was calculated as follows:

(2) $$E_{\text{rel}}=\frac{I_{A}}{\left(I_{D}+I_{A}\right)}$$

with I_A_ = maximum acceptor emission intensity at ∼620 nm, and I_D_ = maximum donor emission intensity at ∼535 nm.

## Results

We wanted to establish an easy-to-use system for manipulating the concentration of sterols in preexisting liposomes and proteoliposomes. m*β*CD is an excellent tool for delivering sterols to model membranes and for removing them (34,37). However, once m*β*CD and (proteo)liposomes are mixed, it is not trivial to separate them. Typically, this separation involves size-exclusion chromatography or harvesting the (proteo)liposomes by ultracentrifugation, which often is inefficient (38). Even though spin concentrators have been successfully used to overcome this issue (39,40), we wanted to know if m*β*CD used in a dialysis setup can support lipid exchange while retaining (proteo)liposomes confined in a separate compartment. This would make subsequent preparative steps more facile, minimize loss of material, and help monitoring the exchange process even in situations when lipids are transported between donor and acceptor liposomes.

**Figure 1:**
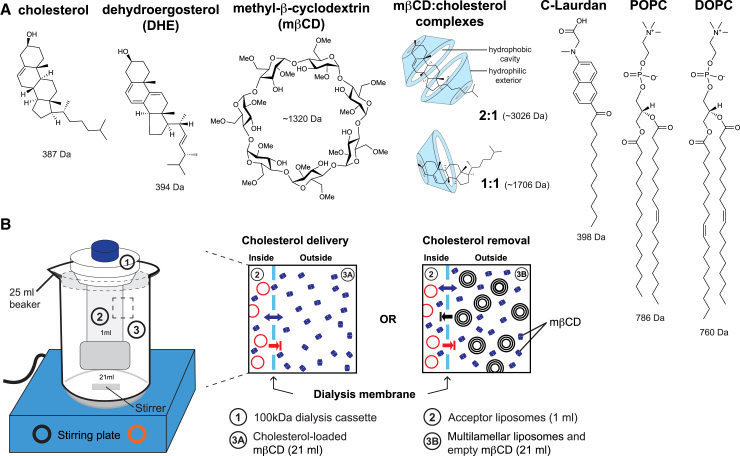
Manipulating cholesterol levels in preformed membranes. (*A*) Chemical structures of molecules used in this study: 1,2-dioleoyl-*sn*-glycero-3-phosphocholine (DOPC), 1-palmitoyl-2-oleoyl-*sn*-glycero-3-phosphocholine (POPC), cholesterol, and dehydroergosterol. Methyl-*β*-cyclodextrin (m*β*CD) is a cyclic oligosaccharide that can bind sterols in different stoichiometries. C-Laurdan is a solvatochromic probe that reports on the degree of water penetration into a lipid bilayer. (*B*) A dialysis cassette with a molecular-weight cutoff of 100 kDa (1) is filled with a 1 mL suspension of either liposomes (200 nmol lipids) or proteoliposomes (600 nmol lipids) (2). The dialysis cassette is placed in outer bath (21 mL) containing either 2.4 mM cholesterol-loaded m*β*CD for cholesterol delivery or 4.8 mM empty m*β*CD with an excess of multilamellar vesicles (400 nmol lipids) as a sink for cholesterol removal. Alternative setups are conceivable. M*β*CD can pass the dialysis membrane.

We used both empty and cholesterol-loaded m*β*CD (Fig. 1 *A*), which can cross a dialysis membrane with a molecular-weight cutoff of 100 kDa (pore size ≈10.5 nm) (Fig. 1 *B*). We hypothesized that it is possible to either deliver or remove cholesterol from preexisting (proteo)liposomes by dialyzing against either cholesterol-loaded or empty m*β*CD (Fig. 1 *B*). Because the (proteo)liposomes are trapped within the cassette, this procedure can be repeated several times. Hence, this experimental setup provides a means to reversibly manipulate the cholesterol concentration in preformed (proteo)liposomes to modulate lipid packing and membrane compressibility.

### Kinetics of cholesterol delivery through a dialysis membrane

**Figure 2:**
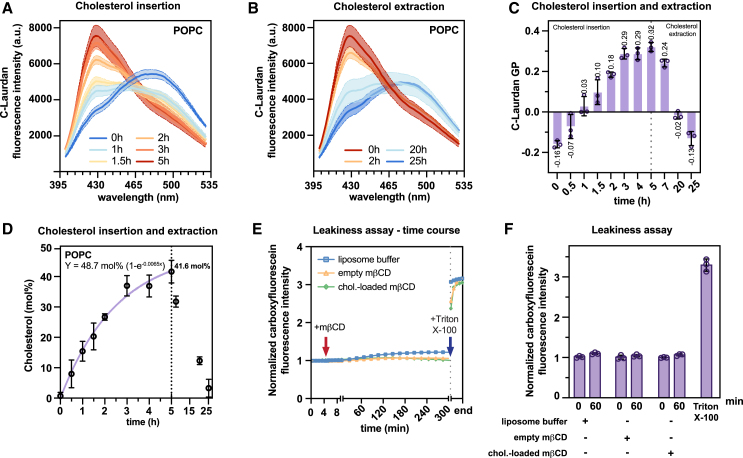
POPC-based liposomes remain intact during cholesterol insertion and removal. (*A*) C-Laurdan fluorescence emission spectra (Ex., 375 ± 5 nm; Em. slit width, 5 nm) of extruded, POPC-based liposomes (200 nmol lipids) after various times of cholesterol delivery. M*β*CD was removed by dialysis before the spectroscopic characterization. Data are from three independent experiments (*n* = 3; mean ± SD). (*B*) C-Laurdan fluorescence emission spectra liposomes (initially 120 nmol) upon cholesterol removal using 4.8 mM empty m*β*CD and multilamellar vesicles (400 nmol) in the outer bath (*n* = 3; mean ± SD). M*β*CD was removed by dialysis before fluorescence spectroscopy. (*C*) C-Laurdan GP values of extruded POPC liposomes during a cholesterol delivery and removal experiment performed at 23°C ± 1°C. The vertical, dotted line indicates the transfer of the dialysis cassette into a new bath and a switch from cholesterol insertion to cholesterol extraction (*n* = 3; mean ± SD). (*D*) Cholesterol concentration (in mol %) derived from the GP values in (*C*) (*n* = 3; mean ± SD). The purple line shows the fit of the experimental data using a one-phase association model (Prism 10) with a fixed plateau of 56.3 mol % and expressing the time in minutes. (*E*) Representative fluorescence emission (Ex., 492 ± 10 nm; Em., 517 ± 10 nm) traces of CF-loaded, POPC-based liposomes (4 nmol of lipids) in a total volume of 200 *μ*L over time at 30°C. After 5 min of equilibration at 23°C ± 1°C (*red arrow*) the extruded liposomes were treated with 0.88 *μ*L of 220 *μ*M cholesterol-loaded m*β*CD (*green*), 440 *μ*M empty m*β*CD (*yellow*), or liposome buffer (*blue*). The addition of Triton X-100 (final concentration 0.1% (w/v)) (*blue arrow*) solubilizes the membrane and releases CF. (*F*) Comparison of the CF fluorescence emission before and after a 60-min dialysis with buffer, 220 *μ*M cholesterol-loaded m*β*CD, or 440 *μ*M empty m*β*CD. The CF emission was normalized to the maximally recorded emission after solubilization by Triton X-100. Data are derived from three independent experiments (*n* = 3; mean ± SD).

First, we were interested in the delivery of cholesterol to liposomes composed of 1-palmitoyl-2-oleoyl-*sn*-glycero-3-phosphocholine (POPC) featuring one saturated and one monounsaturated lipid acyl chain (18). Cholesterol was delivered to liposomes in the cassette (1 mL) using 2.4 mM cholesterol-loaded m*β*CD in the outer bath (24 mL). Cholesterol incorporation was probed at various time points via C-Laurdan spectroscopy (29,41) (Fig. 2 *A*). C-Laurdan is a solvatochromic probe that reports on the degree of water penetration into the lipid bilayer, which is directly related to the inter-lipid spacing (29,41,42). The GP of C-Laurdan is a ratiometric value derived from the fluorescence emission spectrum. It can assume values from +1 (being most ordered) to −1 (being least ordered), but its absolute value depends on the instrumentation and many other factors (41). Due to its carboxylic group, C-Laurdan is partially ionized and flips only slowly across a lipid bilayer. Previously, giant unilamellar vesicles formed from POPC featured a C-Laurdan GP of −0.29, whereas the inclusion of 40 mol % cholesterol resulted in a GP of 0.27, indicative of tighter lipid packing (41). Because C-Laurdan can bind m*β*CD in the absence of liposomes (Fig. S1 *A* and *B*) and because m*β*CD affects the C-Laurdan GP of liposome-containing samples (Fig. S1 *C*), we dialyzed all samples for 19 h against a 125-fold volume and diluted the resulting sample to lower the m*β*CD concentration before C-Laurdan fluorescence spectroscopy. Efficient removal of m*β*CD was confirmed by thin-layer chromatography experiments (Fig. S1 *D*). Upon cholesterol delivery across a dialysis membrane, we observed a dramatic change of the C-Laurdan fluorescence emission spectrum within a few hours (Fig. 2 *A*). Hence, cholesterol-loaded m*β*CD can readily pass the dialysis membrane and unload its cargo into the membrane of acceptor liposomes. Notably, the GP values did not differ when individual experiments were performed for each time point or when several time points were taken from one and the same sample (60 *μ*L each) (Fig. S1 *E*). Assuming no extraction of glycerophospholipids by m*β*CD, only 120 nmol of glycerophospholipids (58% of the total input) are left in the cassette at the end of cholesterol delivery (Fig. 2 *C* and *D*) due to multiple sampling.

Next, we tested if cholesterol can be extracted from liposomes in a dialysis setting. Upon placing the cassette with cholesterol-containing, POPC-based liposomes (120 nmol glycerophospholipid in 580 *μ*L) in a new bath containing 4.8 mM empty m*β*CD and a 3.3-fold excess of POPC-based multilamellar vesicles (400 nmol) as a sink for cholesterol, we observed changes in the fluorescence emission spectrum consistent with a near-complete cholesterol removal (Fig. 2 *B*).

With respect to the C-Laurdan GP values, we observed an increase from −0.16 to 0.32 upon cholesterol delivery (Fig. 2 *C*) and a decrease back to −0.13 upon cholesterol removal (Fig. 2 *C*). Even though C-Laurdan reports predominantly on lipid packing in the outer leaflet of the liposomes, we expect that the obtained spectra are representative for both leaflets, because cholesterol is a fast-flipping lipid that equilibrates between membrane leaflets within seconds (43,44). The observed changes of the C-Laurdan GP suggested an efficient modulation of cholesterol in the liposome membrane. To precisely determine its concentration, we performed a calibration experiment (Fig. S1 *F*). We generated a series of POPC-based, extruded liposomes containing different cholesterol concentrations, recorded C-Laurdan fluorescence emission spectra, and plotted the experimentally determined GP values against the known cholesterol concentration under identical conditions (Fig. S1 *F*). The observed dependency of the GP values from the cholesterol concentration were fitted with a polynomial function, thereby providing a means to deduce the molar concentration of cholesterol based on a C-Laurdan fluorescence emission spectrum (Fig. S1 *F*; supplementary datasheet). Making use of this calibration, we found that the cholesterol concentration in POPC-based liposomes reaches 41.6 mol % within 5 h of delivery with an approximated initial rate of 0.284 ± 0.017 mol % min^−1^ using a mono-exponential association function and its derivative (Fig. 2 *D*; supplementary datasheet). The plateau of this fit was fixed to 56.3 mol %, which was determined in independent experiments long-term cholesterol delivery experiments (Fig. S1 *G*). Because we found no evidence for a change of cholesterol in acceptor liposomes between 24 and 30 h of delivery in these experiments (Fig. S1 *G*), we assumed that an equilibrium was established when cholesterol reaches 56.3 mol % in the membrane (Fig. S1 *G*; supplementary datasheet). Upon placing cholesterol-containing liposomes after 5 h of delivery (Fig. 2 *D*) in a new dialysis bath with empty m*β*CD and multilamellar vesicles as a sink, the cholesterol concentration could be reduced back to 3.3 mol % in a single round of dialysis (Fig. 2 *D*). Hence, it is possible to reversibly tune the cholesterol concentration of liposomes in a dialysis setting.

### Liposomes remain intact during cholesterol exchange

When used in excess, m*β*CD can dissolve liposomes (24,45,46). Even though a lipid:m*β*CD ratio of 1:11 in the inner compartment is lower than the 1:100 ratio normally required for solubilization (46,47), we wanted to verify that our extruded liposomes remain intact during cholesterol delivery as glycerophospholipids may be extracted by m*β*CD after cholesterol unloading and cross the dialysis membrane (Fig. 2 *E* and *F*). To this end, we generated POPC-based liposomes loaded with a self-quenching concentration of CF. CF is a water-soluble, charged fluorophore that is only poorly fluorescent at high concentrations due to excimer formation (48). Hence, for as long as CF is contained in the liposomes, we expect the sample to exhibit only a low level of fluorescence emission. Consequently, membrane rupture or solubilization would lead to an unquenching of CF and a marked increase in the fluorescence intensity. We recorded the CF emission during the incubation of the respective liposomes with either cholesterol-loaded or empty m*β*CD in real time (Fig. 2 *E*). During 5 h of incubation, we observed only minor changes of the fluorescence emission (Fig. 2 *E* and *F*) compared to the threefold increase observed upon solubilizing the liposomes with the detergent Triton X-100 (final concentration ≈1.6 mM) (Fig. 2 *F* and *G*). The mild increase of fluorescence observed for a separate control using liposome buffer without m*β*CD was presumably due to differences in the buffer composition and osmolarity. Nevertheless, we conclude that the liposomes remain intact during the incubation both with cholesterol-loaded m*β*CD or empty m*β*CD. This interpretation was corroborated by DLS experiments, which demonstrate that the average diameter of the liposomes (197 ± 1 nm) is barely affected by cholesterol delivery (200 ± 1 nm) and subsequent cholesterol removal (200 ± 2 nm) (Fig. S1 *H–J*). Somewhat confusingly, we observed that the size distribution was wider after cholesterol delivery (Fig. S1 *H–J*). The basis for this increased heterogeneity of liposome sizes after cholesterol delivery remains unclear and should be investigated in the future by experiments with single liposome resolution. Expectedly, the DLS experiments confirmed that Triton X-100 solubilizes liposomes, because detergent-treated suspensions contained only objects with a diameter of ∼13 nm, which likely corresponds to mixed micelles (Fig. S1 *K*). Together, our experiments suggest that preformed liposomes remain intact when their cholesterol level is remodeled using m*β*CD.

### Cholesterol delivery is affected by lipid packing

Next, we performed cholesterol delivery and removal experiments with extruded liposomes containing an equimolar mix of POPC and DOPC (Fig. S2 *A–E*). Consistent with the acyl chain composition and a lower degree of lipid packing, we also observed lower C-Laurdan GP values for DOPC:POPC-based liposomes (Fig. S2 *A–C*) than for POPC-based liposomes (Fig. 2 *A–C*). Again, we confirmed that the sampling (either each time point individually or several time points from the same sample) had no impact on the observed GP values (Fig. S2 *D*). Thus, we could use a calibration curve to determine the cholesterol concentration based on the C-Laurdan GP (Fig. S2 *E*; supplementary datasheet). Upon dialysis of liposomes with either cholesterol-loaded or empty m*β*CD, we observed either efficient cholesterol delivery or removal, respectively (Fig. S2 *A–C*). Within 5 h of delivery, the cholesterol concentration reached 46.5 mol % (Fig. S2 *A–C*). The initial rate of cholesterol delivery k = 0.360 ± 0.002 mol % min^−1^ (Fig. S2 *F*; supplementary datasheet) was ∼1.3-fold faster compared to the rate observed for more tightly packed POPC liposomes (Fig. 2 *D*), whereas the estimated plateau of 58.4 ± 1.4 mol % (determined in separate long-term delivery experiments) was almost identical for the two lipid environments (Fig. S2 *G*; supplementary datasheet). Our findings suggest that the delivery of cholesterol is faster in a more loosely packed membrane and that DOPC and POPC have similar capacities to incorporate cholesterol (43,49,50,51,52,53,54).

### Modulating the cyclodextrin-dependent cholesterol exchange kinetics

**Figure 3:**
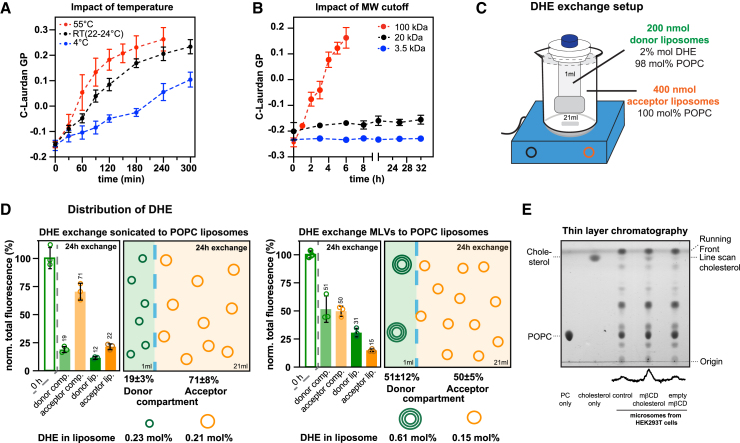
Cholesterol insertion into liposomes is modulated by temperature and the molecular-weight cutoff of the dialysis membrane. (*A*) C-Laurdan GP values of POPC-based liposomes during 5 h of cholesterol delivery at 4°C (*blue*), RT (23°C ± 1°C) (*black*), and 55°C (*red*) using 2.4 mM cholesterol-loaded m*β*CD. M*β*CD was not removed from the sample before C-Laurdan spectroscopy. All spectra were recorded at 30°C. Data are from three independent experiments (*n* = 3; mean ± SD). (*B*) C-Laurdan GP values of extruded liposomes composed of 50 mol % POPC and 50 mol % DOPC upon m*β*CD-mediated cholesterol delivery across dialysis membranes with different molecular-weight cutoffs: 3.5–5 kDa (*blue*); 20 kDa (*black*), and 100 kDa (*red*). M*β*CD was dialyzed out before C-Laurdan spectroscopy. Data are derived from three independent experiments (*n* = 3; mean ± SD). (*C*) Schematic representation of a DHE transfer experiment from donor to acceptor liposomes. (*D*) Either sonicated liposomes (*upper graph*) or multilamellar vesicles (*bottom graph*) composed of 98 mol % POPC and 2 mol % were used as donor liposomes and dialyzed in the presence of empty m*β*CD against a twofold excess of POPC-based liposomes (acceptor liposomes) using a dialysis cassette with a 100-kDa molecular-weight cutoff. (*D*) The transfer of DHE from donor liposomes (200 nmol) to acceptor liposomes (400 nM) at 23°C ± 1°C was followed using the DHE fluorescence emission (Ex, 324 ± 4 nm; Em, 394 ± 4 nm). Either tip-sonified small unilamellar liposomes (*top*) or multilamellar vesicles (*bottom*) were used as donor liposome (200 nmol lipid). The outer batch contained extruded acceptor liposomes (400 nmol lipid) and m*β*CD at an initial concentration of 7.14 mM. The distribution of the normalized total DHE fluorescence across the 1-mL donor and the 21-mL acceptor compartment could be sampled directly, whereas the liposome-associated fluorescence could be determined only after removing m*β*CD-associated DHE by two rounds of dialysis against a 300-fold volume of liposome buffer. The molar concentration of DHE in the donor and acceptor liposomes was calculated assuming an equal distribution of DHE in all membrane leaflets. The data are derived from three independent experiments (*n* = 3; mean ± SD). (*E*) Cholesterol incorporation by m*β*CD into complex membranes was characterized by TLC using chloroform:methanol:H_2_O (70:25:2). 1 mL of crude microsomes isolated from HEK293T cells (0.65 mM lipids) were dialyzed in the presence of membrane buffer 2 (control), cholesterol-loaded m*β*CD (m*β*CD cholesterol) (2.4 mM) or empty m*β*CD (empty m*β*CD) (2.4 mM) for 24 h at 4°C. Bulk m*β*CD was dialyzed out against a 100-fold excess of buffer to lower the m*β*CD concentration. The membranes were sedimented by centrifugation (10,000 × *g*, 10 min, 4°C) subjected to lipid extraction. 330 nmol of lipids were spotted on a TLC silica plate with cholesterol (65 nmol) and POPC (PC) (21 nmol) loaded as a reference. A line scan generated using Fiji indicates the signal intensities caused by cholesterol (36).

Next, we explored how temperature and the molecular-weight cutoff of the dialysis membrane affects the rate of sterol transfer. Initially, we performed the delivery of cholesterol into POPC-based acceptor liposomes at three different temperatures (4°C, 22°C–24°C, and 55°C) and across dialysis membranes with a 100-kDa molecular-weight cutoff (Fig. 3 *A*). For each temperature, we followed the C-Laurdan GP values after various times of cholesterol delivery. Notably, m*β*CD was not removed by dialysis in this experiment and only diluted 4.16-fold before fluorescence spectroscopy. As m*β*CD has an impact on the C-Laurdan GP of extruded liposomes (Fig. S1 *C*), which would cause an underestimation of the cholesterol concentration when using a calibration curve (Fig. S1 *F*), we used the C-Laurdan GP as an indirect measure for cholesterol exchange. Expectedly, the GP increased gradually with cholesterol delivery for all temperatures but more dynamically at higher temperatures (Fig. 3 *A*). Fitting these data to a one phase exponential association model and using a GP value of 0.34 as plateau, we found an impact of temperature on the rate of the GP increase (Fig. 3 *A*; supplementary datasheet): For the delivery at 55°C we obtained a threefold faster initial rate (k_GP,55°C_ = 0.0030 min^−1^) than for the delivery at 4°C (k_GP, 4°C_ = 0.0010 min^−1^).

Similarly, we tested how the molecular-weight cutoff affects the rate of cholesterol delivery to liposomes (50 mol % POPC and 50 mol % DOPC) by following C-Laurdan GP values over time as a proxy for the cholesterol concentration (Fig. 3 *B*). We tested three different dialysis membranes with molecular-weight cutoffs of 3.5, 20, and 100 kDa (Fig. 3 *B*). Compared to the rapid delivery across membranes with a 100-kDa molecular-weight cutoff (pore size of 6–10 nm), the delivery was dramatically slower across membranes with 20- and 3.5-kDa molecular-weight cutoffs as judged from the observed changes of the GP value over time (Fig. 3 *B*). The most obvious explanation is the reduced pore size, which is only 3–5 nm and 1–2 nm for membranes with molecular-weight cutoffs of 20 and 3.5 kDa, respectively. Thus, the passage of m*β*CD across the dialysis membrane can become rate limiting for cholesterol delivery. This is important to consider when choosing the right dialysis membrane for a particular experiment. For example, a membrane with lower pore sizes could be used to slowly “ramp up” the cholesterol concentration in the (proteo)liposomes of interest. Another example could be a m*β*CD- and dialysis-based delivery of cholesterol to nanodiscs, which are smaller than liposomes and may require other lower molecular-weight cutoffs for their containment.

### Sterol exchange between donor and acceptor liposomes

Next, we wanted to know if the dialysis setup is suitable to transfer sterols between liposomes. To this end, we followed the concentration of dehydroergosterol (DHE) in donor and acceptor liposomes, which were separated by a dialysis membrane (Fig. 3 *C*). DHE is a fluorescent analog of ergosterol with three conjugated double bonds (Fig. 1 *A*). Over a broad range of concentrations, the fluorescence emission of DHE is proportional to its molar concentration in liposomes (Fig. S3 *A*; supplementary datasheet) even though DHE is known to self-quench at concentrations higher than 10 mol % (55). This allowed us to directly quantify DHE in both compartments (Fig. 3 *D*). Inside the dialysis cassette, we either used tip-sonified, POPC-based small unilamellar vesicles or multilamellar vesicles, each containing 2 mol % DHE (Fig. 3 *C* and *D*). The outer bath was adjusted to 7.14 mM m*β*CD and contained a twofold excess of extruded POPC liposomes as acceptors, which are initially nonfluorescent. Because a suspension with multilamellar vesicles features a smaller surface area than tip-sonified liposomes, and because some DHE is trapped inside the multilamellar vesicles, we expected a more efficient transfer of DHE with the sonified, small unilamellar vesicles.

After 24 h of lipid exchange, we sampled the distribution of the total DHE fluorescence in both the donor and the acceptor compartment and also determined the fraction of liposome-associated fluorescence after dialyzing out m*β*CD-associated DHE (Figs. 3 *D* and S3 *C*). The normalized total fluorescence in the acceptor compartment was higher when tip-sonified liposomes were used as donors (71%) compared to the case when multilamellar vesicles served as donor liposomes (50%). Thus, more DHE arrives in the acceptor compartment when small unilamellar vesicles are used as donors. Quantifying the liposome-associated fluorescence allowed for calculating the molar concentration of DHE in both donor and acceptor liposomes. When small unilamellar vesicles were used as donors, the concentration of DHE was almost identical in donor and acceptor liposomes (0.23 mol % and 0.21 mol %, respectively), whereas the transfer of DHE from multilamellar donor vesicles was far from complete (0.61 mol % and 0.15 mol % in donor and acceptor liposomes, respectively). In either case, a substantial fraction of DHE remains associated with m*β*CD, which is readily removed by dialysis. Our data demonstrate a delivery of DHE from donor to acceptor liposomes. The dialysis setup helps monitoring the distribution of DHE not only between the two communicating donor and acceptor compartments but also within the compartments between liposomes and m*β*CD. This will help to optimize the rate of lipid transfer in future experiments.

### Cholesterol delivery to complex biomembranes

Cholesterol is an important modulator of membrane compressibility, which crucially affects membrane protein folding, structure, localization, and function (4,56). We wanted to test if m*β*CD, which is often used to manipulate cholesterol levels in the plasma membrane (37), can be used in a dialysis setting for complex biomembranes such as crude microsomes. To this end, we isolated microsomal membranes from HEK293T cells by differential centrifugation and subjected them a cholesterol delivery procedure. A suspension of microsomal membranes (0.65 mM lipid) was placed in the dialysis cassette (1 mL) and dialyzed at 4°C again membrane buffer (control), cholesterol-loaded m*β*CD (2.48 mM) or empty m*β*CD (2.48 mM) in the outer bath. After 24 h of delivery, bulk m*β*CD was dialyzed out, and the liposomes were harvested by centrifugation. Lipids were extracted, separated by thin layer chromatography using a mobile phase of CHCl_3_:MeOH:H_2_O, and stained with iodine (Fig. 3 *E*). We observed increased cholesterol levels in microsomal membranes treated with cholesterol-loaded m*β*CD.

### Studying the impact of cholesterol on the oligomerization of Ire1

Transmembrane protein reconstitution in sterol-rich membranes is challenging especially when there is a significant hydrophobic mismatch between the protein and the lipid bilayer. Energetic penalties associated with hydrophobic mismatch are higher in sterol-rich membranes (13), thereby lowering the efficiency of transmembrane protein insertion. We wanted to test if our setup can provide a means to modulate sterol content after the formation of proteoliposomes.

We decided to study a model transmembrane protein based on the membrane property sensor Ire1 from *S. cerevisiae*. Ire1 uses a hydrophobic mismatch-based mechanism to sense aberrant stiffening and thickening of the endoplasmic reticulum membrane (ER) (4,30,31). Increased membrane thickness and reduced ER membrane compressibility in cells drives Ire1 into dimers and higher oligomers, which ultimately, triggers the unfolded protein response controlling hundreds of target genes (30,32,57,58). Previously, the impact of the membrane environment on Ire1 dimerization was established by continuous-wave electron-paramagnetic spectroscopy using a spin-labeled minimal sensor protein derived from Ire1 (30). Here, we used FRET to assess the oligomeric state of a similar Ire1-based sensor construct as an alternative readout. This Ire1-based sensor construct consists of an N-terminal MBP, a flexible linker with a tobacco etch virus protease recognition site, and the residues P501 to K570 from Ire1, covering its entire transmembrane region with a functionally relevant amphipathic helix and the short transmembrane helix (30,31). A single cysteine was introduced at the C-terminal end of the construct to facilitate fluorescent labeling by maleimide-based chemistry. To prevent complications from undesired covalent crosslinking of Ire1 the endogenous cysteine 552 in the transmembrane helix was replaced by serine. Previously, it was shown that this mutation does not affect the function of Ire1 (30,31).

We co-reconstituted a ATTO514- and ATTO594-labeled constructs in liposomes composed of 50 mol % POPC and 50 mol % DOPC at a protein-to-lipid ratio of 1:16,000, which should minimize proximity FRET from random encounters of labeled Ire1 molecules in the liposome membrane. The acyl chain composition was chosen to reflect the acyl chain composition of the yeast ER in both length and lipid saturation (58,59). Under these conditions, we expect no membrane-based dimerization of Ire1 and therefore no energy transfer between the two fluorophores beyond the proximity FRET, which is caused by random encounters of the labeled proteins in the membrane. Indeed, upon excitation of the ATTO514-labeled donor at 514 nm, we did not observe an increased emission pf the ATTO594-labeled acceptor construct (Fig. S4 *A*).

**Figure 4:**
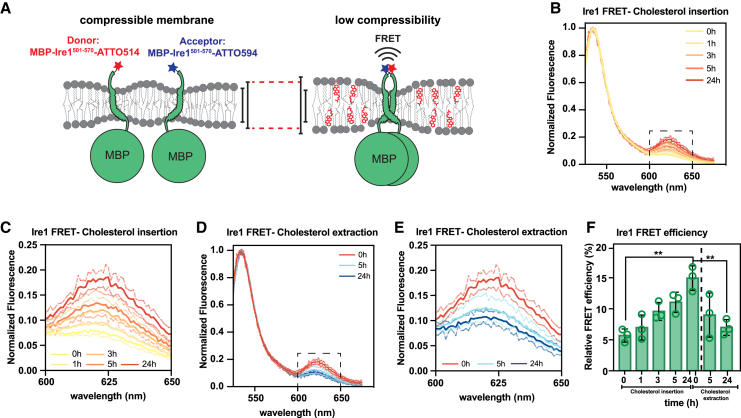
Cholesterol insertion into proteoliposomes induces Ire1 oligomerization in a reversible manner. (*A*) Ire1 oligomerization model in response to lipid bilayer stress and decreased membrane compressibility in vitro. A fusion protein of the maltose-binding protein (MBP) from *E. coli* and the transmembrane domain of the yeast Ire1 (Ire1^aa501-570, C552S^) equipped with a single, C-terminal cysteine was purified and modified with NEM, ATTO514, or ATT594. The proteins were reconstituted in liposomes composed of 50 mol % POPC and 50 mol % DOPC at an overall protein:lipid ratio of 1:16,000 (compressible membrane). Cholesterol delivery to these proteoliposomes lowers the membrane compressibility and increases membrane thickness, which drives Ire1 into oligomers. (*B*) Fluorescence emission spectra were recorded upon donor excitation (Ex., 514 ± 3 nm; Em., 525–675 nm; Em. slit width, 3 nm) and normalized to the maximal donor emission at indicated times during cholesterol delivery via cholesterol-loaded m*β*CD. Spectra are plotted as the mean of three independent reconstitutions (*n* = 3; mean ± SD). (*C*) Normalized fluorescence emission demonstrating FRET at several times over the course of a 24-h cholesterol delivery experiment. (*D*) Fluorescence emission spectra were recorded upon donor excitation (Ex., 514 nm; Em., 525–675 nm; slit width, 3 nm) and normalized to the maximal donor emission at indicated times during 24 h of cholesterol extraction via empty m*β*CD. Spectra are plotted as the mean of three independent reconstitutions (*n* = 3; mean ± SD). (*E*) Normalized fluorescence emission showing the FRET shoulder at the indicated times during 24 h of cholesterol extraction using empty m*β*CD. (*F*) The relative FRET efficiency was derived from the fluorescence spectra in (*B*) and (*D*) and plotted as mean of three independent reconstitutions (*n* = 3; mean ± SD). A two-tailed, unpaired *t*-test was performed to test for statistical significance (^∗^*p* < 0.05, ^∗∗^*p* < 0.01, ^∗∗∗^*p* < 0.001).

When we used our dialysis setup to deliver cholesterol to the FRET pair-containing proteoliposomes using m*β*CD, we observed over time increasing FRET signals (Fig. 4 *B* and *C*), suggesting an increased proximity of the donor and the ATTO594-labeled acceptor constructs is likely caused by the dimerization of Ire1. No such changes in the fluorescence spectrum were observed in proteoliposomes containing the ATTO514-labeled donor alone (Fig. S4 *C*). The increased FRET signal upon cholesterol delivery to FRET pair-containing liposomes was abolished, when the proteoliposomes were solubilized with detergents (Fig. S4 *B–D*). We conclude that successful cholesterol delivery triggers a change in membrane properties that induces dimerization/oligomerization of Ire1-derived sensor constructs.

In contrast to classical reconstitution schemes, our m*β*CD-based transfer of cholesterol also allows for removing cholesterol from proteoliposomes. This reversibility also provides a means to distinguish between a functional, cholesterol-triggered oligomerization and an irreversible aggregation of a transmembrane protein from an unsuccessful reconstitution. After cholesterol was delivered to proteoliposomes containing Ire1 constructs forming a FRET pair (Fig. 4 *D*, 0 h), we removed it again by placing the dialysis cassette in a new bath containing empty m*β*CD and multilamellar vesicles as a sink. The removal of cholesterol from the proteoliposomes reestablishes membrane compressibility and causes the dissociation of Ire1 dimers. Indeed, the relative FRET efficiency was substantially lower after 5 h or cholesterol removal and even more so after 24 h (Fig. 4 *D* and *E*). Hence, using a dialysis-based sterol exchange setup combined with FRET, we demonstrate the potential of this approach to manipulate the behavior of reconstituted transmembrane proteins in a controlled and reversible manner. Tuning sterol levels and membrane compressibility in preformed proteoliposomes could become a useful and widely used tool to study the impact of the lipid bilayer on membrane protein structure, dynamics, and function.

## Discussion

We have implemented an easy-to-use experimental setup to reversibly modify sterol levels in preexisting (proteo)liposomes. Because dialysis does not require expensive instrumentation, this approach is broadly accessible to virtually every biophysical, pharmaceutical, or biochemical laboratory. Sterol transfer to and from proteo(liposomes) is mediated by m*β*CD shuttling between two compartments separated by a dialysis membrane. Throughout sterol exchange, the (proteo)liposomes are retained in their compartment, thereby facilitating easy recovery, straightforward buffer exchange, and quantitative m*β*CD removal whenever necessary. Our proof-of-principle experiments with liposomes and proteoliposomes show that cholesterol delivery increases lipid packing (Figs. 2 and 3) and decreases membrane compressibility as suggested by the membrane-driven dimerization of a membrane property sensor module derived from Ire1 (Fig. 4) (4,30,31,32).

The dialysis setup is versatile and provides several advantages: 1) membrane material can be recovered with excellent yields. 2) Donor and acceptor liposomes remain separated throughout the experiment, thereby preventing undesired membrane fusion and facilitating a parallel, spectroscopic characterization of both samples (Fig. 3 *D*). 3) The concentration of sterols can be adjusted after the formation of proteoliposomes, whereas it is often challenging to yield sterol-rich environments for a membrane protein using standard reconstitution procedures. 4) The gradual delivery of sterol is both time- and cost-effective: a single transmembrane protein reconstitution in a sterol-free membrane provides sufficient material for a whole set of samples covering a broad range of sterol concentrations and membrane compressibilities (Fig. 4). 5) The versatile dialysis setup is likely applicable not only to liposomes but also to nanodiscs (60), natural and synthetic exosomes (61,62), and complex ER-derived microsomes (Fig. 3 *E*), thereby widening the scope of potential applications.

Nevertheless, the dialysis-based setup has limitations, and not all possible applications have been explored. 1) Lipid delivery to liposomes (Figs. 2 and 3) and proteoliposomes (Fig. 4) takes hours. Only sufficiently stable transmembrane proteins that survive the time of dialysis can be suitably studied using this approach. 2) The specificity of m*β*CD does not guarantee the exclusive transfer of sterols, and some level of glycerophospholipid transfer cannot be ruled out. In fact, m*β*CD is known to bind and exchange glycerophospholipids at high concentrations (34,45,63,64,65), but less so at the concentrations used in this study. Furthermore, m*β*CD has different affinities for different types of lipids (24), thereby further complicating the challenges when working with complex lipid mixtures such as biomimetic membranes. Thus, it will be important to quantify lipid recovery especially when handling (proteo)liposomes with complex lipid compositions. In this context, it may be informative to investigate the heterogeneity of (proteo)liposomes using total internal reflection fluorescence microscopy (15) or emerging technologies such as mass spectrometry imaging (66). 3) Although it is theoretically useful for generating asymmetric liposomes, the m*β*CD-mediated transfer of glycerophospholipids is too slow under the conditions established here (data not shown). A significant portion of glycerophospholipids delivered to the outer leaflet of an acceptor (proteo)liposome would passively flip to the luminal leaflet in the time of dialysis.

One of the key challenges in studying the structure, dynamics, and function of transmembrane proteins in complex, native-like membrane environments arises from the heterogeneous distribution of membrane components in (proteo)liposomes, which can be accentuated by the presence of sterols (15,19,20,21,22). The m*β*CD-mediated delivery of sterols to preexisting (proteo)liposomes, which is best accomplished in a dialysis setup, provides a potential work-around scheme to potentially overcome at least some of these issues. Because sterol delivery is reversible, we can distinguish between irreversible aggregation and reversible, membrane-based oligomerization as demonstrated for the sensory module of the membrane property sensor Ire1 (Fig. 4).

We are convinced that this setup for lipid exchange can be applied to both model membranes and biomembranes by providing an on-demand delivery of sterols and/or phospholipids. After manipulation of the lipid composition, individual (proteo)liposome fractions can be removed from the dialysis setup and subjected to virtually any type of biophysical analysis, including fluorescence spectroscopy, CD spectroscopy, native mass spectrometry, dynamic light-scattering, electron-paramagnetic resonance spectroscopy, and cryo-electron microscopy. Hence, the dialysis-based setup for a m*β*CD-mediated lipid transfer will be a valuable tool to characterize the structure and function of membrane proteins in different lipid environments.

## Acknowledgments

The authors wish to thank Alexander von der Malsburg for critical reading of the manuscript and John Reinhard for fruitful discussions in the early phase of the project. We a particularly grateful to Toni Radanović for his meticulous efforts in establishing and optimizing the reconstitution of Ire1 in liposomes. This work was funded by the start-up funding for research ideas by the Research Committee of the 10.13039/501100005690Saarland University to C.A., the 10.13039/501100001659Deutsche Forschungsgemeinschaft in the framework of the SFB1027 to R.E., and by the 10.13039/501100000781European Research Council under the European Union’s Horizon 2020 research and innovation program (grant agreement no. 866011) to R.E.

## Author contributions

C.A. designed experiments, provided supervision, performed research, analyzed data, and wrote the first draft of the manuscript. E.R. performed research and analyzed data. J.H. performed research, analyzed data, and provided technical and administrative support. M.F.R. contributed analytical tools and performed research. R.E. designed research, provided supervision, analyzed data, and wrote the manuscript.

## Declaration of interests

The authors declare no competing interests.

## Supporting material

Document S1. Figures S1–S4

Data S1. Supplementary datasheet

Document S2. Article plus supporting material
