# Supplementary material for: Reversible tuning of membrane sterol levels by cyclodextrin in a dialysis setting
Source: Biophys J. 2025 Mar 25;124(9):1433–45. doi: 10.1016/j.bpj.2025.03.020 (PMC12256852; doi:10.1016/j.bpj.2025.03.020)
Supplement: Document S1. Figures S1–S4 [file mmc1.pdf]

Biophysical Journal, Volume *124*

Supplemental information

Reversible tuning of membrane sterol levels by cyclodextrin in a dialysis setting

Cynthia Alsayyah, Emmanuel Rodrigues, Julia Hach, Mike F. Renne, and Robert Ernst

Supplementary Material

Reversible tuning of membrane sterol levels by cyclodextrin in a dialysis setting

Cynthia Alsayyah[1,2,3], Emmanuel Rodrigues[1,2,3], Julia Hach[1,2,3], Mike F. Renne[1,2,3] & Robert Ernst[1,2,3*]

[1] Medical Biochemistry and Molecular Biology, Medical Faculty, Saarland University, Homburg (Saar), Germany

[2] Preclinical Center for Molecular Signaling (PZMS), Medical Faculty, Saarland University, Homburg (Saar), Germany

[3] Center for Biophysics (ZBP), Saarland University, Saarland

*Corresponding author: robert.ernst@uni-saarland.de

(Supplementary Figures S1-4)

Figure S1

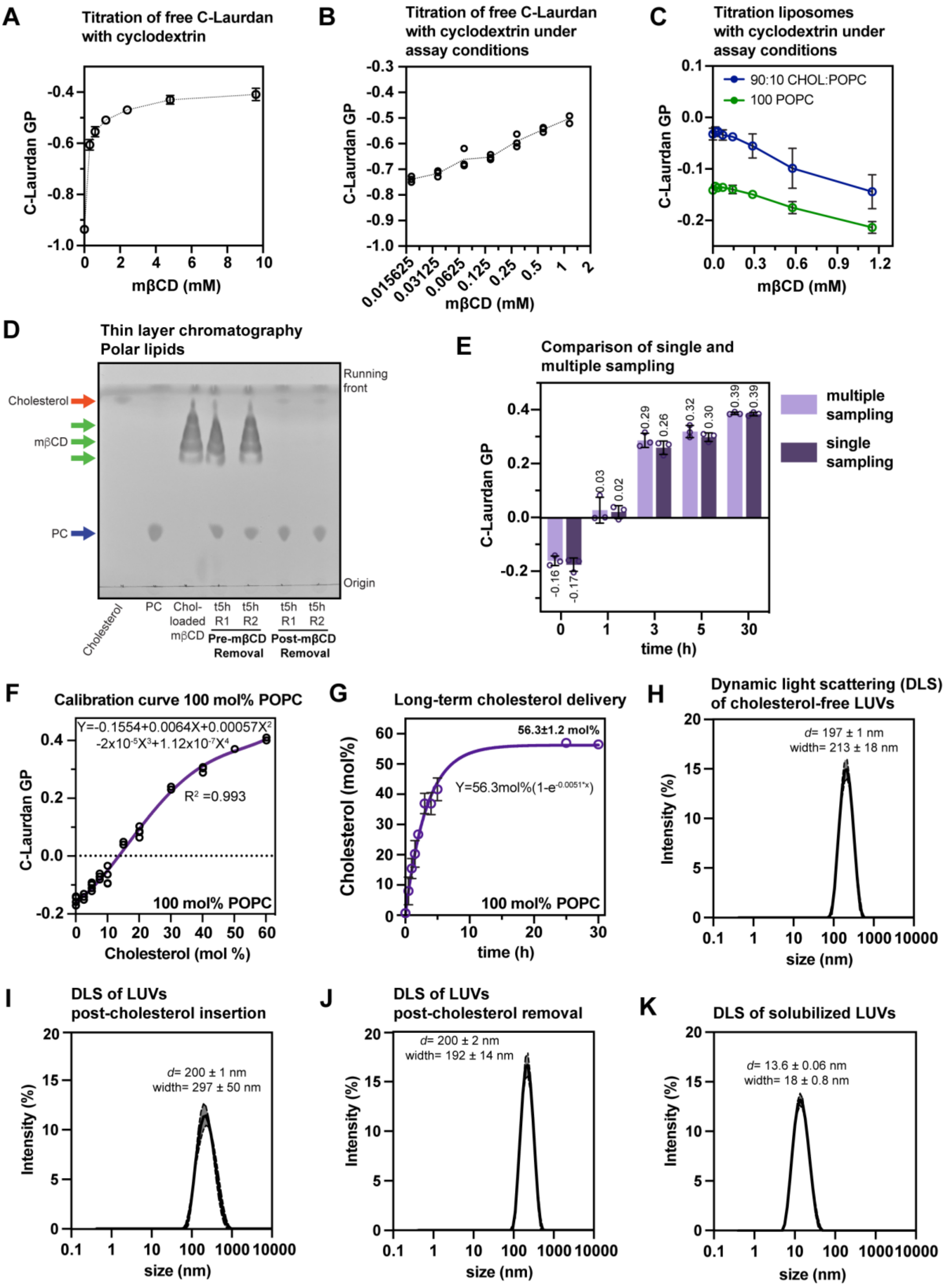

A
Titration of free C-Laurdan with cyclodextrin
C-Laurdan GP
mβCD (mM)
B
Titration of free C-Laurdan with cyclodextrin under assay conditions
C
Titration liposomes with cyclodextrin under assay conditions
90:10 CHOL:POPC
100 POPC
D
Thin layer chromatography Polar lipids
Cholesterol
mβCD
PC
Running front
Origin
Chol-loaded mβCD
t5h R1
t5h R2
Pre-mβCD Removal
Post-mβCD Removal
E
Comparison of single and multiple sampling
multiple sampling
single sampling
time (h)
F
Calibration curve 100 mol% POPC
Y=-0.1554+0.0064X+0.00057X²-2x10⁻⁵X³+1.12x10⁻⁷X⁴
R² =0.993
100 mol% POPC
Cholesterol (mol %)
G
Long-term cholesterol delivery
56.3±1.2 mol%
Y=56.3mol%(1-e^-0.0051*x)
Cholesterol (mol%)
H
Dynamic light scattering (DLS) of cholesterol-free LUVs
d= 197 ± 1 nm
width= 213 ± 18 nm
Intensity (%)
size (nm)
I
DLS of LUVs post-cholesterol insertion
d= 200 ± 1 nm
width= 297 ± 50 nm
J
DLS of LUVs post-cholesterol removal
d= 200 ± 2 nm
width= 192 ± 14 nm
K
DLS of solubilized LUVs
d= 13.6 ± 0.06 nm
width= 18 ± 0.8 nm

Figure S1: (A) The impact of mβCD on the C-Laurdan GP was determined in the absence of liposomes at 23±1°C. The fluorescence emission of C-Laurdan (0.1 µM) was recorded in 20 mM HEPES pH 7.4, 150 mM NaCl, 5% glycerol (w/v) at different concentrations of mβCD (Ex.: 375±5 nm; Em. slit width: 5 nm). The resulting GP values are plotted against the mβCD concentration. **(B)** The impact of mβCD on the C-Laurdan GP was determined in the absence of liposomes after a 4.16-fold dilution to mimic the assay conditions used in cholesterol delivery and removal experiments. The concentrations on the Xaxis correspond to the final concentration of mβCD after dilution. **(C)** The impact of mβCD on the C-Laurdan GP in the presences of POPC-based liposomes either with 10 mol% cholesterol (blue) or without (green). Liposomes (200 µM total lipid) were adjusted to different mβCD concentrations up to 4.8 mM and incubated for 5 min at 30°C. To mimic the assay conditions for cholesterol delivery and removal, 60 µl of the liposome suspension were diluted 4.16-fold and adjusted to 0.1 µM C-Laurdan before the emission spectrum was recorded. The concentrations on the X-axis correspond to the final concentration of mβCD after dilution. **(D)** The efficiency of mβCD removal by dialysis under assay conditions was studied by TLC. POPC-based liposomes (20 nmol lipid) were loaded onto the silica plate. Cholesterol (65 nmol lipid), POPC (PC) (21 nmol lipid), and Cholesterol-loaded mβCD (0.28 µmol) were used as standards. Notably, cholesterol cannot be resolved in the presence of mβCD for the replicates R1 and R2 (Post-mβCD removal). However, after removal of mβCD by dialysis against a 125-fold volume for 19 h, cholesterol becomes discernable for both replicates (Post-mβCD removal). **(E)** The C-Laurdan GP of extruded POPC-based liposomes subjected to a 30-hour cholesterol delivery procedure with 2.4 mM cholesterol-loaded mβCD in the outer bath. The influence of single sample versus multiple sampling (removing 60 µl for each time point from the dialysis cassette) was investigated. Prior to C-Laurdan spectroscopy, mβCD was dialyzed out (n=3)**. (F)** The C-Laurdan GP of POPC-based liposomes containing different concentrations of cholesterol was determined and used to generate a calibration curve. A polynomial function was used to fit the experimental data as indicated using a fixed plateau of 56.3 mol%. Each data point represents one of three independent experiments (n=3). **(G)** Cholesterol concentration in POPC-based liposomes derived from a standard curve (n=3; mean±SD) at different times during a long-term cholesterol delivery experiment. The green solid line shows the fit of the experimental data using a one phase association model (Prism 10) with a fixed plateau of 56.3 mol% and using the time of delivery in minutes. **(H-K)** The intensity-weighted particle size distribution of POPC-based liposomes was determined by DLS. Three technical replicates from a single liposome preparation are shown. The average particle size (or Z-average) is indicated as (*d*±SD) as well as the full width at half maximum (width±SD). The individual samples are **(H)** liposomes prior to cholesterol delivery, **(I)** after 5 h of cholesterol delivery, **(J)** after subsequent cholesterol removal, and **(K)** after complete solubilization through the addition of with Triton X-100.

Figure S2

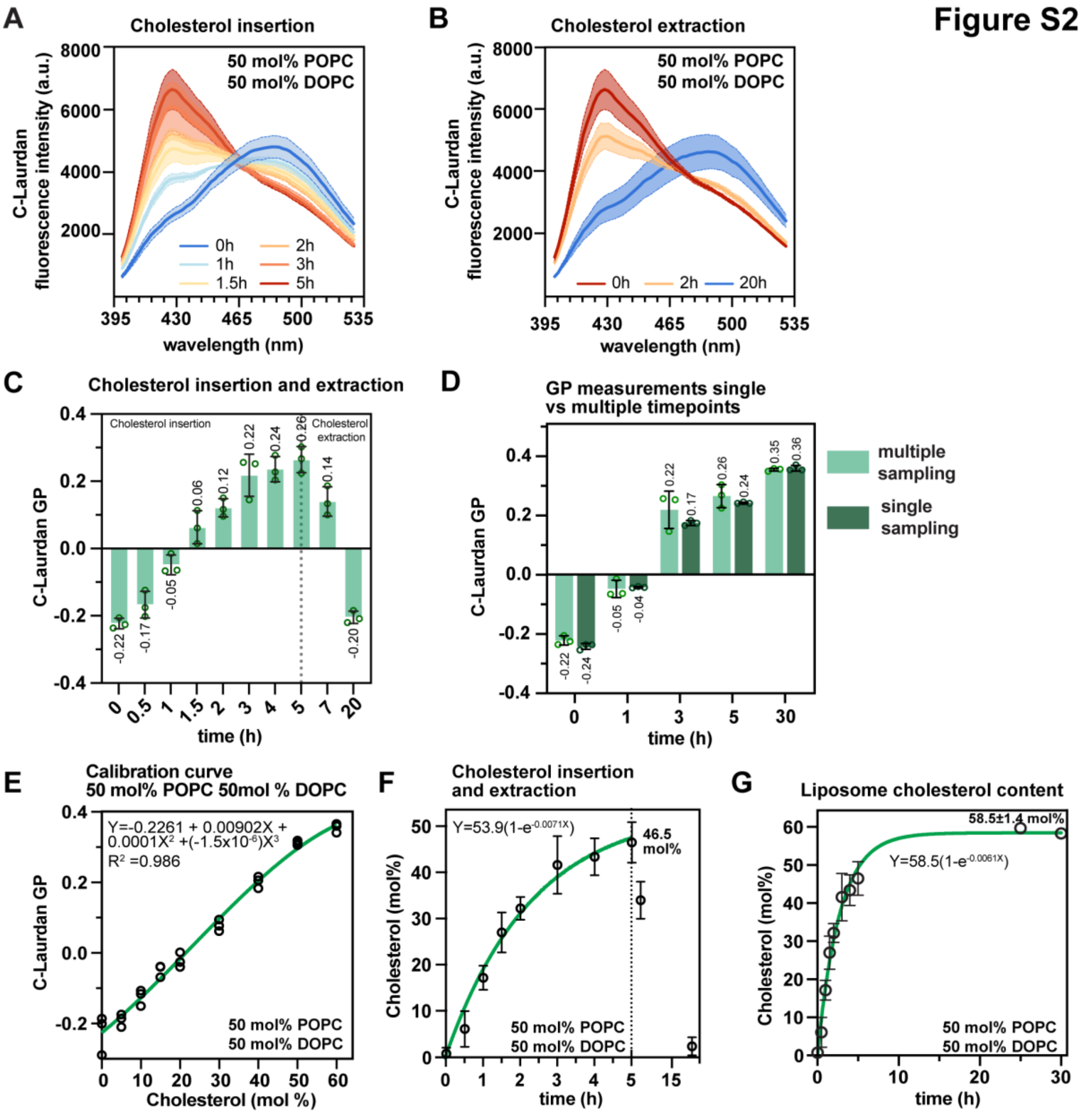

A
Cholesterol insertion
50 mol% POPC
50 mol% DOPC
C-Laurdan fluorescence intensity (a.u.)
wavelength (nm)
0h 1h 1.5h 2h 3h 5h
B
Cholesterol extraction
50 mol% POPC
50 mol% DOPC
0h 2h 20h
C
Cholesterol insertion and extraction
Cholesterol insertion
Cholesterol extraction
C-Laurdan GP
time (h)
D
GP measurements single vs multiple timepoints
multiple sampling
single sampling
C-Laurdan GP
time (h)
E
Calibration curve
50 mol% POPC 50mol % DOPC
Y=-0.2261 + 0.00902X + 0.0001X² +(-1.5x10⁻⁶)X³
R² =0.986
C-Laurdan GP
Cholesterol (mol %)
50 mol% POPC
50 mol% DOPC
F
Cholesterol insertion and extraction
Y=53.9(1-e^-0.0071X)
46.5 mol%
Cholesterol (mol%)
time (h)
50 mol% POPC
50 mol% DOPC
G
Liposome cholesterol content
58.5±1.4 mol%
Y=58.5(1-e^-0.0061X)
Cholesterol (mol%)
time (h)
50 mol% POPC
50 mol% DOPC

Figure S2: Cholesterol delivery and removal to and from extruded liposomes composed of equimolar mix of POPC and DOPC. **(A)** POPC:DOPC-based liposomes (200 nmol lipids) were dialyzed against 21 ml of 2.4 mM cholesterol-loaded mβCD. At indicated times, an aliquot was removed and the C-Laurdan fluorescence emission spectrum was recorded (Ex.: 375±5 nm; Em. slit width: 5 nm). Data are from three independent experiments (n=3; mean±SD). **(B)** C-Laurdan fluorescence emission spectra of cholesterol-loaded liposomes (initially 120 nmol glycerophospholipids) upon cholesterol removal using empty mβCD and extruded POPC:DOPC liposomes (400 nmol lipids) in the outer bath of the dialysis setup. Data are from three independent experiments (n=3; mean±SD). **(C)** C-Laurdan GP of extruded liposomes composed of 50 mol% POPC and 50 mol% DOPC during the exchange at 23±1°C. The dotted vertical line indicates the transfer of the dialysis cassette from a cholesterol delivery to cholesterol removal setting after five hours of delivery. Shown are independent triplicates (n=3; mean±SD). (**D**) The C-Laurdan GP of extruded POPC:DOPC-based liposomes subjected to a 30-hour cholesterol delivery procedure using 2.4 mM cholesterol-loaded mβCD. The influence of single sample versus multiple sampling (removing 60 µl from the dialysis cassette for each time point) investigated. Prior to C-Laurdan spectroscopy, mβCD was dialyzed (n=3). **(E)** The C-Laurdan GP of POPC:DOPC-based liposomes containing different concentrations of cholesterol was determined and used to generate a calibration curve. A polynomial function was used to fit the experimental data as indicated. Each data point represents one of three independent experiments (n=3). (**F**) Cholesterol concentration (in mol%) in liposomes derived from a standard curve was determined for different samples during cholesterol delivery. The green solid line represents a fit to the experimental data using a one phase association model (Prism 10) with a fixed plateau of 58.4 mol% and using the time of delivery in minutes. Data are from three independent experiments (n=3; mean±SD). **(G)** Cholesterol concentration (in mol%) in extruded POPC:DOPC-liposomes derived from a standard curve (n=3; mean±SD) was determined for different samples during cholesterol. The green solid line represents a fit to the experimental data using a one phase association model (Prism 10) and using the time of delivery in minutes. The fit was used to approximate the equilibrium concentration of cholesterol in the liposome membrane (58.4 mol%).

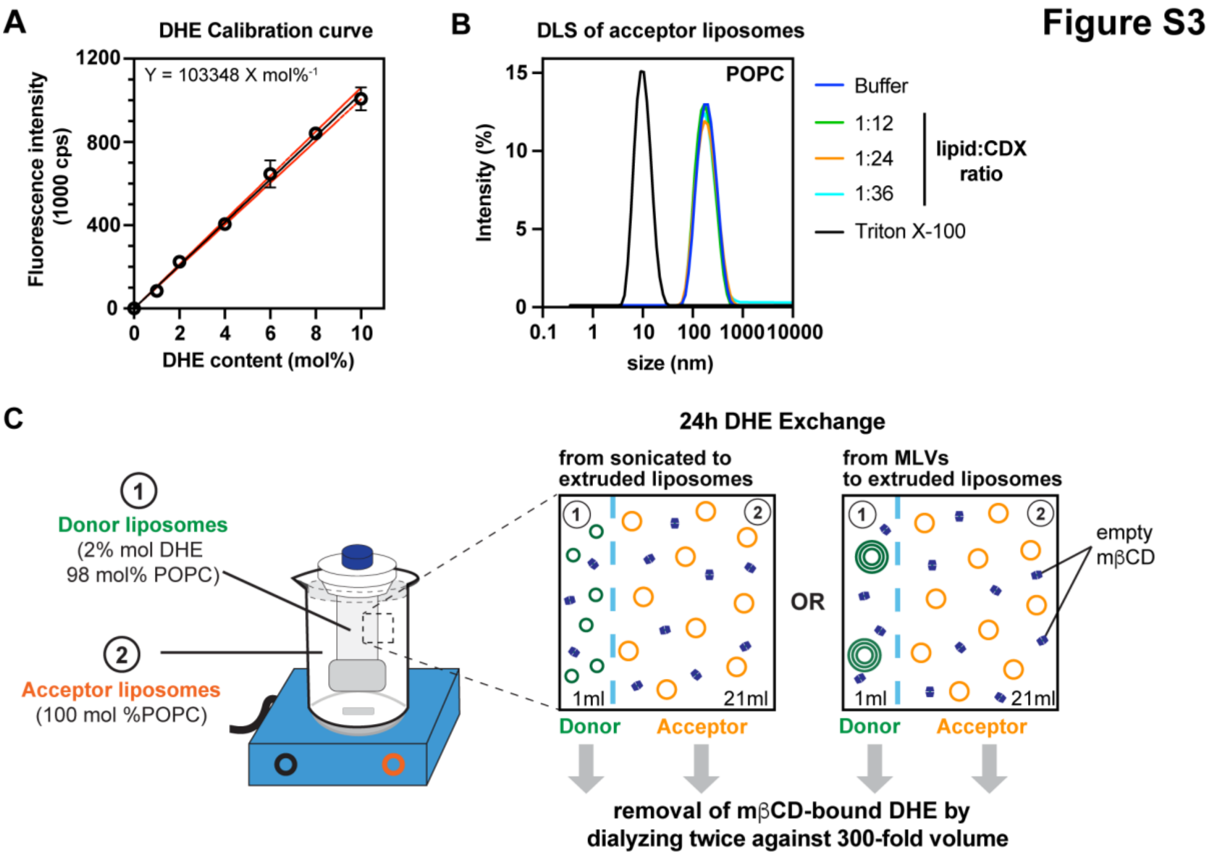

Figure S3: DHE calibration series for DHE content calculation. **(A)** A linear dependency of the DHE fluorescence emission (Ex.: 324±4 nm; Em: 394±4 nm) from the DHE concentration in POPC-based, extruded liposomes (1.1 nmol lipid in 120 µl) was observed. The solid black line represents a linear regression forced through the origin. The data are derived from three independent experiments (n=3; mean±SD). **(B)** The intensity-weighted particle size distribution of POPC-based liposomes (2 nmol lipid in 100 µl) was determined by DLS. The experimental conditions and the lipid:mβCD ratio have no apparent impact on the size of extruded liposomes. In the presence of Triton X-100, liposomes are solubilized. **(C)** The transfer of DHE from donor liposomes (200 nmol) to acceptor liposomes (400 nM) at 23±1°C was followed using the DHE fluorescence emission (Ex: 324±4 nm; Em: 394±4 nm). Either tip-sonified small unilamellar liposomes (top left panel) or multilamellar liposomes (top right panel) were used as donor liposomes. Following a 24 hours exchange between donors and acceptors in a light-protected environment, the mβCD-associated DHE was removed from the liposome-associated DHE by dialyzing the donor compartment twice against a 300-fold volume of liposome buffer. Likewise, 1 ml of the acceptor compartment was dialyzed twice against a 300-fold volume of liposome buffer to remove mβCD-associated DHE.

Figure S4

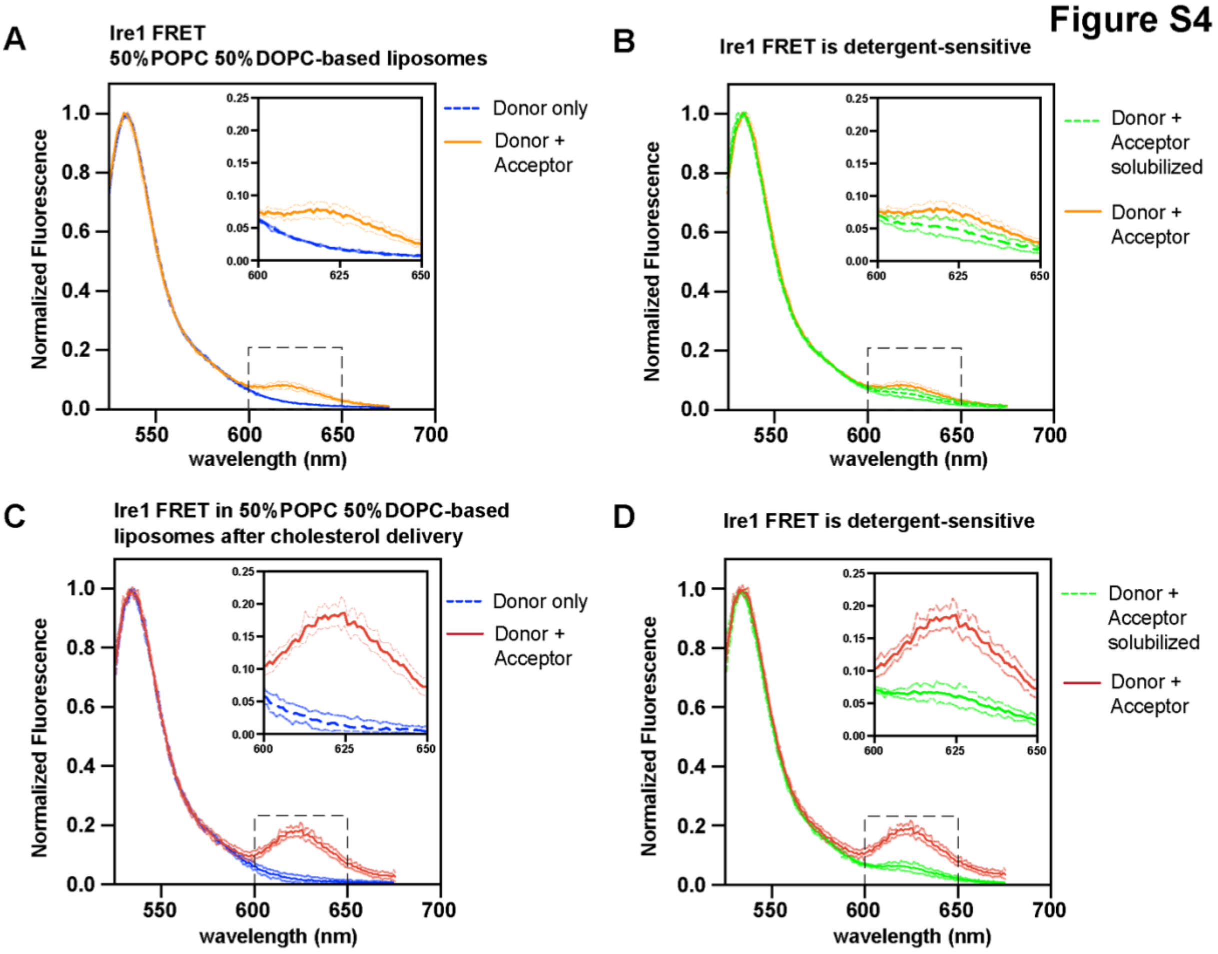

Figure S4: Validating the impact of cholesterol on the oligomerization of Ire1. Fluorescence emission spectra of the indicated samples were recorded upon excitation of the donor (ATT514) fluorophore (Ex.: 514±4 nm; Em.: 525–675 nm; Em. slit width: 3 nm) and normalized to the maximal donor emission. (**A**) Comparison of the fluorescence emission spectrum of proteoliposomes containing only donor-labeled MBP-Ire1$^{aa501\text{-}570,\ C552S}$ or a FRET pair. (**B**) Comparison of the fluorescence emission spectrum before and after solubilization of the proteoliposomes (20 mM HEPES pH 7.4, 150 mM NaCl, 7% (w/v) glycerol, 50 mM OG, 4 mM SDS). (**C**) Comparison of the fluorescence emission spectrum of proteoliposomes containing only donor-labeled MBP-Ire1$^{aa501\text{-}570,\ C552S}$ or a FRET pair after 24 hours of cholesterol delivery. (**D**) Proteoliposomes with a FRET pair show increase acceptor emission after 24 hours of cholesterol delivery. Upon solubilization of the proteoliposomes with detergent (20 mM HEPES pH 7.4, 150 mM NaCl, 7% (w/v) glycerol, 50 mM OG, 4 mM SDS), no acceptor fluorescence can be observed. All spectra are plotted as the mean of three measurements from independent reconstitutions (n=3; mean±SD).
